# Evolution of Structural and Functional Diversity of Spexin in Mammalian and Non-mammalian Vertebrate Species

**DOI:** 10.3389/fendo.2019.00379

**Published:** 2019-06-19

**Authors:** Chor Hong Lim, Megan You Min Lee, Tomoko Soga, Ishwar Parhar

**Affiliations:** Brain Research Institute, Jeffrey Cheah School of Medicine and Health Sciences, Monash University Malaysia, Bandar Sunway, Malaysia

**Keywords:** neuropeptide, galanin receptor, reproduction, fat metabolism, obesity

## Abstract

Spexin (SPX) is a novel neuropeptide, which was first identified in the human genome using bioinformatics. Since then, orthologs of human SPX have been identified in mammalian and non-mammalian vertebrates. The mature sequence of SPX, NWTPQAMLYLKGAQ, is evolutionally conserved across vertebrate species, with some variations in teleost species where Ala at position 13 is substituted by Thr. In mammals, the gene structure of SPX comprises six exons and five introns, however, variation exists within non-mammalian species, goldfish and zebrafish having five exons while grouper has six exons. Phylogenetic and synteny analysis, reveal that SPX is grouped together with two neuropeptides, kisspeptin (KISS) and galanin (GAL) as a family of peptides with a common evolutionary ancestor. A paralog of SPX, termed SPX2 has been identified in non-mammalians but not in the mammalian genome. Ligand-receptor interaction study also shows that SPX acts as a ligand for GAL receptor 2 (2a and 2b in non-mammalian vertebrates) and 3. SPX acts as a neuromodulator with multiple central and peripheral physiological roles in the regulation of insulin release, fat metabolism, feeding behavior, and reproduction. Collectively, this review provides a comprehensive overview of the evolutionary diversity as well as molecular and physiological roles of SPX in mammalian and non-mammalian vertebrate species.

## Introduction and Background

Using bioinformatics search method based on the Hidden-Markov model, two independent groups identified spexin (SPX, also known as NPQ) (1, 2). The sequence of the mature peptide of SPX is constituted of 14 amino acids, flanked by dibasic cleavage sites, which are evolutionally conserved in vertebrates. Due to its high conservation across species, SPX is predicted to be involved in diverse and important biological functions (1). SPX is present in various brain regions and peripheral tissues, and its functional role has been associated with feeding behavior, obesity, reproduction, cardiovascular, and nociception (3). Among these, the most prominent and emerging role of SPX has been linked to the regulatory role of metabolic homeostasis. In 2010, a microarray study revealed that SPX was significantly down-regulated in the omental and subcutaneous fat of obese human subjects (4). This has prompted the authors to conduct a functional study in rodents, exhibiting the ability of SPX to reduce fatty acids uptake and also to induce weight loss in obese rodents (5). Since then, a large body of experimental evidence has accumulated that is in line with this observation. Although these functional studies were largely conducted in mammals, recent studies have also examined genome and physiological role in non-mammalian vertebrates, in particular, the fish species (6, 7). A major discovery that SPX can act as a ligand for GAL receptor type 2 and 3 has advanced the understanding of signaling mechanism of SPX (8). This review takes a comparative approach to outline various aspects of SPX in the vertebrate species.

## Structure and Evolution of Spexin

### Structure

**Figure 1:**
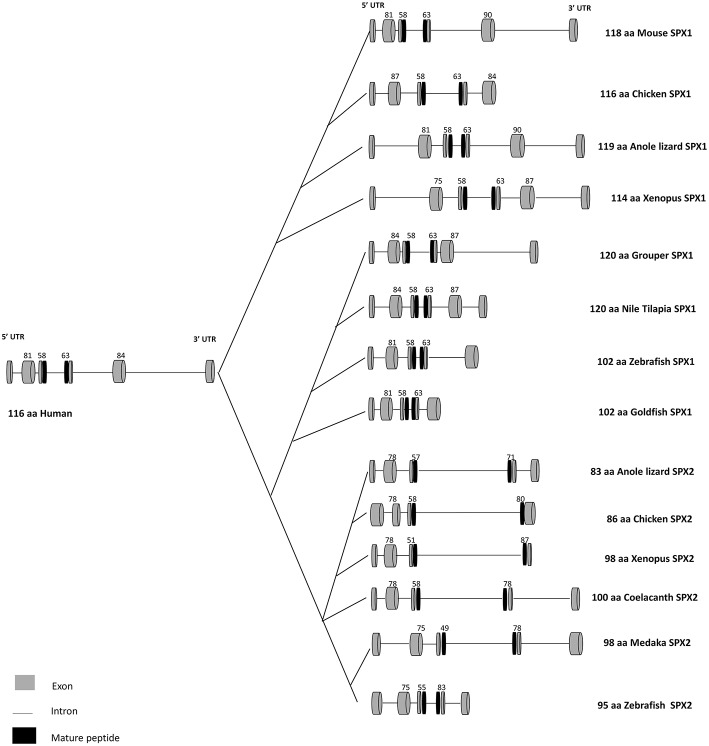
Gene structure of SPX1 and SPX2 in vertebrates. Comparison of SPX1 and SPX2 gene structure in vertebrates across species. The first and the second exons encode the signal peptide, followed by the third and fourth exons which encode the mature peptide sequence. Goldfish and zebrafish SPX1 genes are composed of five exons and four introns. Modified from Wong et al. (6) and Li et al. (9). The full sequences of SPX genes were downloaded from GenBank and the intron junctions were deduced manually or using Splicing Finder 3.1 and aligned according to the length of the sequence. The gene structure of SPX2 was deduced based on the predicted sequence from GenBank.

**Figure 2:**
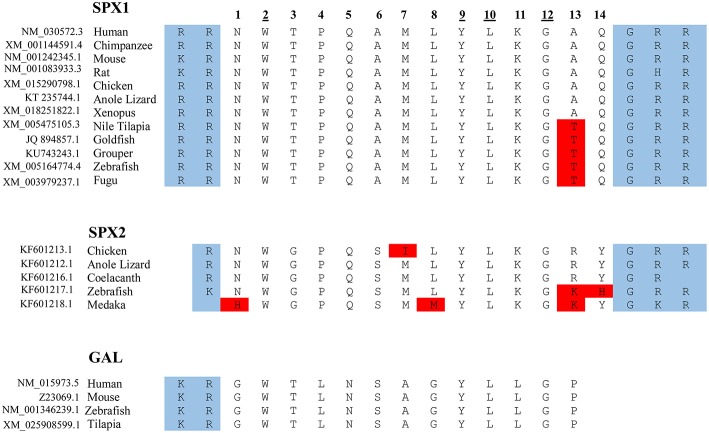
Protein sequence of SPX and GAL. The mature sequence of SPX1 is highly conserved across species, with only slight variation. Note that in SPX1, there is an exception with the cleavage site of the rat, where arginine is replaced with histidine, and GHR instead of GRR. Only a partial sequence of the full mature peptide of GAL is shown here. The amino acid at positions 2, 9, 10, and 12 are conserved across the 3 lineages for SPX1, SPX2, and GAL. The protein cleavage site is boxed in blue and the amino acid substitution is boxed in red.

**Figure 3:**
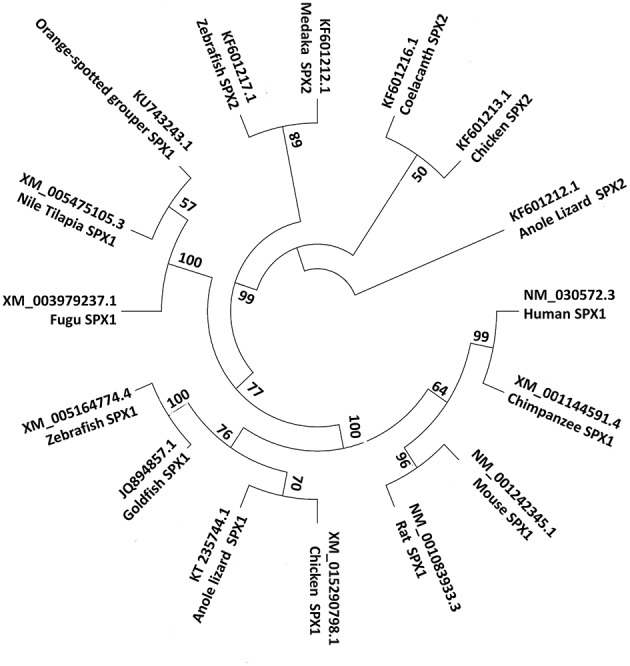
Phylogenetic tree of SPX1 and SPX2. SPX1 is present across all vertebrates, while SPX2 is absent in mammals. For the phylogenetic tree, SPX nucleotide sequences were downloaded from GenBank. The phylogenetic tree was constructed by Mega 7 software using the neighbor joining method. The numbers present on the branch points are percentage scores based on 1,000 bootstraps.

In humans, the gene that encodes SPX is located on chromosome 12, namely C12orf39. SPX gene consists of 6 exons and 5 introns, encoding a prepropeptide of 116 amino acids. In teleosts, the genomic organization of SPX is not conserved. SPX gene in the goldfish and zebrafish is composed of five exons and four introns (6). On the contrary, the genomic structure of SPX in grouper is like the mammalian counterpart, which consists of six exons and five introns (9). A study conducted in the goldfish SPX (6) showed alternative splicing of the intron, producing three mRNA transcripts of different length (847bp, 805bp, and 574bp). However, only the transcript of 574bp carries the mature peptide sequence of SPX, while the other two do not due to the presence of premature stop codon in the intron sequence. Although this observation is reported only in the goldfish, this may hold true for other vertebrate species. Thus, depending on the primer sequence used for analysis, this may produce variation in the results obtained either from quantitative real-time PCR or *in-situ* hybridization. The comparison of SPX gene structure and mature peptide sequences for vertebrate species is illustrated in Figures 1–3.

The primary structure of SPX contains recognizable sequences of signal peptide along with two dibasic pro-hormone cleavage sites. The first and the second exon encode the signal peptide; the third and fourth exons encode the mature peptide sequence. The mature sequence contains 14 amino acids and is flanked by monobasic and dibasic cleavage sites (1). Orthologs of human SPX have been identified in other species which, include chimpanzee, mouse, rat, chicken, anole lizard, Nile tilapia, goldfish, grouper, zebrafish, and fugu (8). This highly conserved sequence across species has slight variation in position 13 (Thr vs. Ala). In teleosts, the amino acid Alanine at position 13 is substituted by Threonine in the mature peptide sequence of SPX. The mature sequence NWTPQAMLYLKGTQ is conserved across the fish species (refer to as the fish SPX) (3).

The 3-D structure of SPX was first described in goldfish by Wong et al. (6). The first 4 amino acids form a random structure in the N-terminus, followed by the remaining amino acids (5 to 14) that form an α-helical structure, which extends to the C-terminus. In the mouse, the 3-D structure of SPX is dominated by an α-helical structure (10). The 3-D conformation is not unique to SPX, similar conformation has been reported in the pituitary adenylate cyclase-activating polypeptide (11), neuropeptide y (12), and vasoactive intestinal peptide (13).

### Evolution and Diversification of Spexin in Vertebrate

The investigation of the evolutionary mechanisms leading to SPX generation was first elucidated by Kim et al. (8). At first, they carried out the synteny analyses for SPX and their neighboring genes on the chromosomes across vertebrates. They found that all SPX, Kisspeptin (KISS) and Galanin (GAL) genes are closely located in a linkage group. In addition, the phylogenetic analysis performed using the mature peptide sequence of SPX, GAL and KISS showed that the SPX family is phylogenetically closer to the GAL family than to the KISS family of genes (8). These three neuropeptides KISS/GAL/SPX have been grouped together as a peptide family that arose from a common ancestor.

Based on synteny analysis and data acquisition, another form of SPX, called SPX2 was found across non-mammalian vertebrate species (8). The initial SPX is now termed SPX1. There are no reports of SPX2 in mammals; perhaps it is absent in the mammalian genome (Figure 2). SPX2 shares a similar coding exon structure with SPX1, where monobasic and dibasic cleavage sites flanking the mature sequence. However, the mature peptide of SPX2 is bordered by Arg (R) and Gly-Arg-Arg (GRR)/ Gly-Lys-Arg (GKR), and variation exists at positions 1, 3, 6, 7, 13, and 14. (8). Despite the variation in the mature sequence of SPX1 and SPX2, the mature sequence is highly conserved across species, suggesting that both SPX1 and SPX2 play important physiological role in vertebrate species.

## Receptor and Distribution of Spexins in Vertebrates

### Receptor of Spexins

From a molecular evolution perspective SPX has some degree of similarity with GAL (8). In SPX1, the amino acids at positions 2, 3, 9, 10, and 12 are identical to the corresponding position in GAL (Figure 2). This similarity in sequence is the key determining factor for the interaction between SPX and galanin receptor (GALR), because amino acids in positions 2, 3, and 9 (correspond to the amino acid Trp^2^, Thr^3^, Tyr^9^) in GAL are the determining criteria for the receptor binding and activation (14). Mammals have three subtypes of GALR, namely GALR1,GALR2, and GALR3. In non-mammalian vertebrates, there are two paralogs of GALR1 (GALR1a and GALR1b) and GALR2 (GALR2a and GALR2b). However, GALR3 is absent in the teleost genome (3, 8). Syntheny analysis conducted by Kim et al. (8) showed that mammalian GALR1 is similar to non-mammalian GALR1a and mammalian GALR2 is classified as the same class as GALR2a in non-mammalian vertebrates.

**Figure 4:**
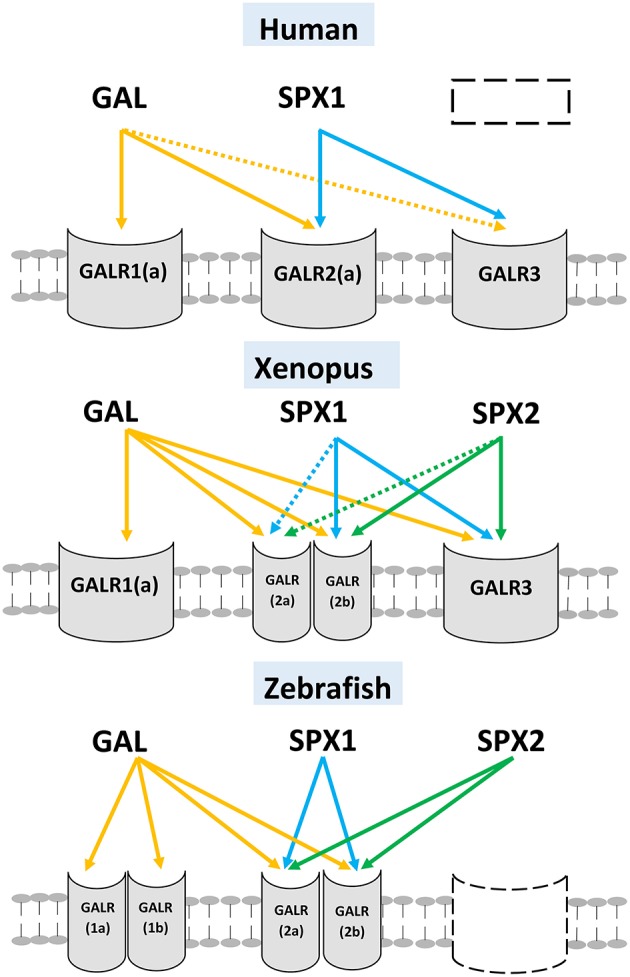
Ligand-receptor interaction between SPXs and different subtypes of GALR. Three different vertebrate's classes (mammals, amphibian, and teleost) are illustrated here. GAL can activate all subtypes of GALR but exhibits low affinity toward GALR3. SPX1 and SPX2 can activate both GALR2 (2a and 2b) and GALR3, but fail to activate GALR1 (1a and 1b). The gene/receptor that are absent in the genome of the vertebrate are represented by dash box). The interaction is illustrated based on the work of Kim et al. (8). (Dotted arrow: Low affinity).

A ligand-receptor study conducted across vertebrate species showed that SPX1 and SPX2 can activate galanin receptor 2 (2a and 2b) and 3, but not galanin receptor 1 (1a and 1b) (8). In the Xenopus and zebrafish, both SPX1 and SPX2 exhibits higher potency toward GALR2b than GAL (8). These studies reveal that SPX is a natural ligand for GALR2 and GALR3. The ligand-receptor interaction between SPX and GAL is illustrated in Figure 4.

#### Diversity in Distribution of GALR2/3

In the brain of mammals, GALR2a positive cells are localized in the mammillary, arcuate, and supraoptic nuclei of the hypothalamus, the dentate gyrus and CA3 of the hippocampus, and also the spinal trigeminal tract, dorsal vagal complex in the hindbrain [reviewed in (15)]. Another study showed that GALR3 positive cells are visualized in the dorsomedial, ventromedial and paraventricular nuclei of the hypothalamus, regions of the forebrain (the medial amygdaloid nucleus, the diagonal band of Broca, bed nucleus of the stria terminalis, and the medial septum), the PAG in the midbrain, and to also the lateral parabrachial nucleus of the hindbrain (16). GALR3 positive cells are also identified in the subfornical organ and medial medullary reticular formation. There are several regions where GALR2a and GALR3 overlap, which includes the ventral tegmental area, substantia nigra, nucleus accumbens, and locus coeruleus.

There is limited information available about the distribution of GALR in teleosts. In the Atlantic salmon and the sea bass, using autoradiography, GAL binding sites, which may indicate the presence of GALR (mostly GALR1a) were observed in the telencephalon, forebrain bundles, the sublayers of the optic tectum, torus semicircularis, dorsolateral thalamic nucleus, hypothalamus, raphe nuclei, pituitary, ventral medulla oblongata, and the dorsal spinal cord (17, 18). In a recent study in the zebrafish, GALR2a has been localized in a restricted area in the ventral telencephalon/subpallium, while the distribution of GALR2b positive cells has been reported in the posterior tuberculum, olfactory bulb, dorsal thalamus, midbrain tegmentum, hindbrain, preoptic region, post-optic commissure and spinal cord (19).

The absence of GALR3 but the presence of two GALR type 2(GALR2a /2b) in teleost, suggests a possible conservation and diversity of these two receptors in their functional role, as seen for the sea bass GALR2a/2b. Comparing the amino acid sequence of teleost GALR2a/2b with that of human GALR2a, the physico-chemical characteristics of both the N- and C- terminus are well-conserved in GALR2a/2b, although teleost GALR2a has shorter terminus than the human GALR2a (20). For ligand binding activity both GALR2a/2b receptors retain some key amino acids identified for receptor binding in human GALR2a (His252, His253, Phe264, and Tyr271), with the exception of GALR2b where His252 is substituted with a tyrosine residue (20). In addition, the N-terminus is also important for ligand binding (21, 22). Indeed, GALR2b has evolutionally highly conserved N- and C-terminal and key amino acid essential for ligand binding than GALR2a. The wide distribution and highly conserved gene structure of GALR2b suggest that GALR2b is the important receptor for spexins in teleosts (20).

#### Mechanism of Spexin Synthesis, Regulation, and Release

Classical peptide hormones are synthesized in the endoplasmic reticulum, followed by transportation to the Golgi apparatus and packed into secretory granules to be released when stimulated. Mirabeau et al. (1) employed the anti-FLAG immunocytochemistry method and transfected rat pancreatic cells with FLAG-SPX, and found FLAG-labeled SPX co-localized with insulin in a small punctate located in the cytoplasm. Similarly, Wan and colleagues employed the anti-Myc method and transfected with COS-7 cells and detected the Myc-tagged SPX in the extracellular medium. These studies suggest that the synthesis and release mechanism of SPX is similar to other typical peptide hormones. However, both anti-FLAG and anti-Myc method are not suitable for detection of hydrolyzed and amidated peptides, and therefore a better detection method is needed. To answer this question, Wan and colleagues developed an antibody against SPX and further used it to study the process and secretion of SPX (23).

Most peptide hormones undergo several post-translational modification processes, including amidation to attain its active form. Since SPX contains the typical amidation signal, it was expected that SPX also undergoes amidation before being activated into its active form. Surprisingly, SPX was found to be secreted into the extracellular medium chiefly as a full-length protein, contrary to the expectation of being a hydrolyzed and amidated peptide (23). This was confirmed by Wong et al. (6), who injected both amidated and non-amidated forms of goldfish mature SPX peptides using intraperitoneal (i.p.) and intracerebroventricular (i.c.v.) method in the goldfish and found suppression of food consumption and a concurrent rise in food regurgitation/vomiting. Similarly, both amidated and non-amidated forms of SPX when injected into the goldfish inhibit luteinizing hormone (LH) release (7). This suggests that amidation of the C-terminal may not be essential to the bioactivity of SPX. However, recent finding in a mouse model suggests that amidation process is essential for the functional role of SPX in pain response (24). The discrepancy in the biological activity of amidated vs. non-amidated SPX may be due to differences in the receptors involved in the regulation of LH and modulation of pain response.

### Tissue/Brain Distribution of Spexins

**Figure 5:**
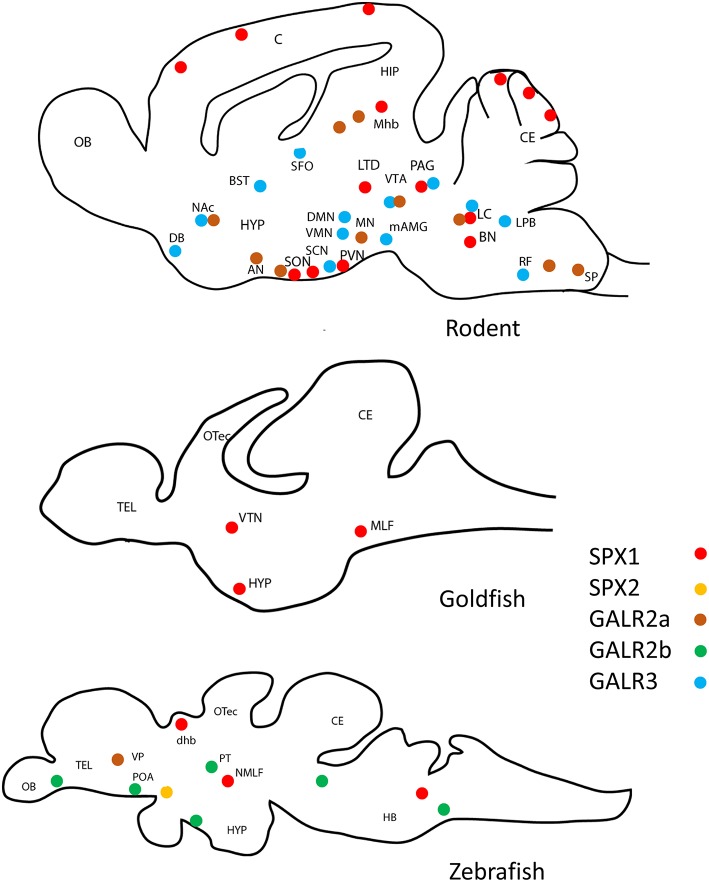
Schematic drawing of the representative localization of SPX1, GALR2a/2b, and GARL3 in the sagittal sections of the brain of various vertebrate species. SPX1 has only localized in rodent (2, 25, 26), goldfish (7), and zebrafish (27, 28). GALR2a/2b was not localized in goldfish. (Red dots represent SPX1 neurons, orange dots represent SPX2 neurons, brown dots represent GALR2a, green dots represent GALR2b, and blue dots represent GALR3. OB, Olfactory bulb; C, cerebral cortex; CE, cerebellum; OTec, optic tectum; Tel, telencephalon; HYP, hypothalamus; SON, supraoptic nuclei; PVN, paraventricular nuclei; LTD, laterodorsal tegmental; PAG, periaqueductal gray; BN, barrington's nucleus; LC, locus coeruleus; VTN, ventromedial thalamic nucleus; MLF, medial longitudinal fasciculus; NMLF, nucleus of the medial longitudinal fasciculus; HB, Hindbrain; POA, preoptic area; dhb, dorsal habenula; Mhb, medial habenula;MN, mammillary nuclei; AN, arcuate nuclei; SON, supraoptic nuclei; DG, dentate gyrus; SP, spinal trigeminal tract; DMN, dorsomedial nuclei, VMN, ventromedial nuclei; PVN, paraventricular nuclei; mAMG, medial amygdaloid nucleus; DB, diagonal band of Broca; BST, bed nucleus of the stria terminalis; LTD, lateral parabrachial nucleus; SFO, subfornical organ; RF, medial medullary reticular formation; VTA, ventral tegmental area; SN, substantia nigra; SCN, suprachiasmatic nucleus NAc, nucleus accumbens; VP, ventral pallium; PT, posterior tuberculum.

Since its discovery in 2007 (1), an extensive analysis of the localization of SPX across species, showed its presence in a wide range of organs and tissues in both mammalian and non-mammalian vertebrates. The distribution of SPX1 and SPX2 in the brain of the rodent, goldfish and zebrafish is illustrated in Figure 5.

### In Mammalian Vertebrates

The discovery of SPX by Mirabeau et al. (1), showed SPX localized in the submucosal layer of esophagus and stomach fundus of the rat. SPX is detected across a wide range of tissues studied, including major systems in the body like respiratory, cardiovascular, skeletal, digestive, excretory, reproductive and endocrine systems. Using *in-situ* hybridization, SPX has been localized in the Barrington's nucleus, in the caudal extent of the mesopontine tegmentum, overlapping closely with corticotrophin-releasing hormone (CRH) (2). In addition, SPX signals are also detected in the locus coeruleus and laterodorsal tegmental nucleus (2). SPX is also observed in the ventrolateral quadrant of the caudal periaqueductal gray (PAG) co-localized with tyrosine hydroxylase and tryptophan hydroxylase 2 (2). Following the work by Sonmez et al. (2), a more detailed examination of SPX localization in the rat was performed by Porzionato et al. (25). In this study, ependymal cells in the choroid plexus show the most intense immunoreaction for SPX, followed by the neurons of the superior cervical, trigeminal ganglia and photoreceptor cells in the retina, and Purkinje cells in the cerebellar cortex. SPX is also observed in the hypothalamic paraventricular and supraoptic nuclei. SPX signals are also seen in the trophoblastic cells of the human term placenta (23). Cortex of adrenal gland in the adult rats (29) and Type I glomic cells within the carotid body of both rat and human (30) are also positive for SPX. Intriguingly, SPX signals was detected in the medial habenula and suprachiasmatic nucleus of the hypothalamus in mice (26).

#### In Non-mammalian Vertebrates

SPX1 and SPX2 have been reported in the genome of various non-mammalian species (Figures 2, 3) such as chicken, anole lizard, and several fish species, however, there is only one available study regarding the distribution and function of SPX2. Therefore, the discussion below will focus mainly on SPX1 in fish species.

#### In Fish Species

In the brain of goldfish, SPX1 is expressed in the optic tectum, hypothalamus, the brain stem, cerebellum, telencephalon, and the pituitary (6). SPX1 positive cells are also reported in the ventromedial thalamic nucleus and medial longitudinal fasciculus of the goldfish (7), and in the zebrafish (27). Recently, Kim et al. (28) reported the detailed distribution of both SPX1 and SPX2 in the brain of the zebrafish. This study is the first to report the localization of SPX2 in non-mammalian vertebrate. SPX1 is localized in the midbrain and hindbrain. Further examination showed SPX1 localized in the dorsal habenula that projects to the interpeduncular nucleus. On the other hand, SPX2 is localized in the hypothalamic preoptic area. The discrepancy in the localization of SPX1 reported by Kim et al. (28) and Zheng et al. (27) may be due to the use of different detection methods; *in-situ* RNA hybridization (28), and immunofluorescence using polyclonal antibody raised against human SPX1 (27).

## Functions of Spexin

The widespread distribution of SPX is consistent with the findings of diverse functions related to metabolism, reproduction, and neuropsychiatric disorder.

### Fat Metabolism and Feeding Behavior

The role of SPX in the regulation of feeding behavior was first shown by Wong et al. (6) using goldfish as an animal model. SPX mRNA levels were elevated following food intake and i.c.v. injection of SPX inhibited feeding behavior induced by orexigenic factors, Neuropeptide Y and orexin. GALR2 receptor has been localized in the supraoptic, paraventricular, and arcuate nuclei of the hypothalamus; these regions have been associated with feeding behavior (31, 32). SPX is significantly decreased in the forebrain of the fasting Ya-fish but increases after refeeding (33). On the contrary, in the orange-spotted grouper, half-smooth tongue sole, and spotted scat, SPX expression increased following fasting (9, 34, 35). In addition, in the orange-spotted grouper (9) and the goldfish (6), i.p. injection of SPX increased gene expression level of anorexigenic factors proopiomelanocortin (POMC) and suppressed the expression of orexigenic factors orexin in the hypothalamus. These studies suggest that SPX acts as a satiety factor, which plays a vital role in the regulation of feeding behavior. Also, SPX gene expression is increased by insulin following food intake in hepatocytes and brain cell cultures from goldfish (36), which suggest a connection between SPX expression and food intake regulated by insulin.

Following the findings of its ability to act as a satiety factor, the functional role of SPX was further examined in obesity/ fat metabolism-related diseases, such as metabolic syndrome and diabetes mellitus (37–39).

In obese patients a significant down-regulation of SPX gene expression is observed in omental and subcutaneous fat (5). Also, circulating SPX levels are significantly lower in obese children (40, 41) and patients with metabolic syndrome (39) or remain unchanged in obese adolescents (42). In addition, Kumar and colleagues reported low level of SPX and high level of leptin in the blood of obese adolescent, together with high level of high sensitivity-C reactive protein (hs-CRP). As high level of hs-CRP is linked to higher risk of cardiovascular disease, this finding suggests a possible role of SPX in cardiovascular risk in obese children (43). There is evidence of an increase in serum SPX levels in severely obese patients following 6 months after the Roux-En-Y gastric bypass surgery and the associated weight loss (44), implying dysregulation of SPX in fat metabolism during obesity.

In diet-induced obese rodents, daily i.p. SPX injections reduce caloric intake with corresponding weight loss (5). Further, *in vitro* study shows adipocytes isolated from obese mouse or healthy human subjects and incubated with SPX significantly impede fatty acid uptake into the adipocytes (5, 45). In addition, SPX down-regulates the expression of pro-adipogenic genes while increases lipolysis by stimulating the phosphorylation of hormone sensitive lipase (45). Incubation of hepatocytes isolated from the Non-alcoholic Fatty Liver Disease (NAFLD) mouse model with SPX directly inhibits uptake of long chain fatty acids (37). SPX treatment *in vivo* for 4 weeks reduces hepatic lipids by 60% (37). i.p. injection of SPX significantly reduces the hepatic and circulating total bile acids level in the rats (46), and this reduction can be halted by GALR2 and GALR3 antagonists (46). These functional studies, as a whole, are in line with the idea that SPX acts as an adipokine, which plays a vital role in the regulation of fat metabolism and therefore a potential target for obesity treatment.

In both type 1 and type 2 diabetes mellitus patients, SPX levels are reduced in serum and negatively correlate with blood glucose levels (47, 48). Further, treatment with SPX to diet-induced obese mice with type-2 diabetes mellitus, improves glucose tolerance and decreases insulin resistance (37). Zheng et al. (27) demonstrated that SPX knockout zebrafish exhibit high appetite and high level of glucose, triacylglycerol and cholesterol in the serum, further proving the essential role of SPX in glucose tolerance and fat metabolism. Similarly, Kolodziejski et al. (49) found low serum levels of SPX and KISS in obese patients and a negative correlation between SPX and KISS with insulin resistance. Recent evidence has also shown that there is a decrease in insulin response to glucose following SPX treatment in pancreatic cells/islets cultured from obese mice (50). This decrease in insulin level is accompanied by increase in cell viability and proliferation of the cultured pancreatic cells. A decrease in insulin release is associated with increased insulin sensitivity, this indicates a potential use of SPX treatment for diabetic or obese patients (50). Indeed, a negative feedback mechanism is observed between SPX and insulin in the pancreatic islets (51). SPX is also elevated in gestational diabetes patients, along with a positive correlation increase in glucose concentration levels while decreased in pregnant women without gestational diabetes (52). In summary, these studies have establish the potential role of SPX as a biomarker of metabolic parameter, in particular insulin resistance.

### Reproduction

In teleosts, SPX suppresses the reproductive axis both *in vivo* and *in vitro* systems (7). i.p. injections of SPX into the goldfish has been shown to induce the suppression of serum LH level and incubation of cultured pituitary cells with SPX also suppresses LH. The expression of SPX mRNA was lower during the breeding season, and the mRNA expression of hypothalamic SPX is regulated by gonadal hormones. SPX is regulated by estrogens since SPX levels increase after ovariectomy and this increase is halted by estrogen replacement (7). Subsequently, SPX was downregulated in the hypothalamus after treatment with estrogen both *in vivo* and *in vitro* (34). Similarly, in the orange spotted grouper (9) hypothalamic expression of SPX is lower during the breeding season but injections of SPX do not influence the expression of LH in the pituitary. In the half-smooth tongue sole, i.p. injection of SPX increased the mRNA expression of gonadotropin-inhibitory hormone and gonadotropin-releasing hormone 3 in the hypothalamus (35). However, knockout of SPX in the zebrafish does not induce any changes (excitatory or inhibitory) on the reproductive capability (27). This can be explained by the complex reproductive mechanism in teleosts. Knockout of a particular neuropeptide does not significantly impact reproduction as there are other functional neuropeptides present that compensate the loss (53). In addition, SPX inhibits the expression of growth hormone both *in vivo* and *in vitro* in orange-spotted grouper and half-smooth tongue sole (9, 35). Although SPX is a cognate ligand for GALR2/3, its biological activity related to reproduction contradicts that of GAL. GAL stimulates the release of LH as opposed to inhibition by SPX (54). This could explain the inhibitory (GALR1/3) or stimulatory (GALR2) effect of the respective receptor on the G-protein signaling pathway (55).

### Other Functions

In cultured adrenal cortical cells in rat, SPX stimulates the release of aldosterone and corticosterone while inhibits the growth of adrenocortical cells (29). The finding of SPX localized in the ventrolateral quadrant of PAG can be hypothesized to be associated with the nociceptive activity (2). Indeed i.v. (intravenous) injection of SPX in the mice produces anti-nociception effect (56) and intrahippocampal CA3 injection of SPX produces a significant decrease in pain sensitivity in ovariectomized rat (57). The authors also highlighted that the anti-nociception effect is even greater when SPX and progesterone are co-administrated. These results suggest that SPX serves as a pain modulator and progesterone enhances the analgesic effect of SPX. SPX mediates its anti-nociception action via GALR3 (24).

In mice, injections of SPX stimulates the intestinal/colonic smooth muscle contractions and enhances the bowel movement via activation of L-type voltage-gated calcium channels (10). Introduction of GALR2 but not GALR3 antagonist suppresses the stimulatory effect of SPX on bowel movement. This suggests that SPX action is mediated via GALR2. SPX is also found in the carotid body, responsible for detecting changes in oxygen levels. Rats exposed to hyperoxia during the first 2 weeks of postnatal life lead to diminution of the size of carotid body, and subsequently, SPX gene expression is up-regulated (30).

SPX also has effects on cardiovascular and renal functions (56). i.c.v. injection of SPX in rat causes an effect similar to the action of angiotensin II, which includes an increase in mean blood pressure and decrease in heart rate and urine flow rate. An i.v. bolus injection of SPX produces an acute pressor and bradycardic response in conscious rats that are dose-dependent but does not have any effect on the urine flow rate. This contrasting result may be due to the rapid metabolism of SPX when injected via i.v. method and thus no effect is seen on the renal function. Recently, in addition to the proposed functional role of SPX in lipid metabolism, glucose homeostasis and insulin resistance, serum level of SPX was found to be decreased with age in healthy women investigated (38). This suggests that SPX could be involved in aging and its related disorders. Of note, the previous finding that GALR2 agonist could exhibit neuroprotective effect on the beta-amyloid toxicity in the rat forebrain (58) provides a glimpse into the potential role of SPX in the pathology of aging related disorders. On the other hand, stepwise regression analysis revealed that SPX is significantly associated with Interleukin-1β level, which suggests a possible role of SPX in immune modulation (59).

## The Potential Role of SPX in Stress, Depression and Anxiety

Activation of GALR2 via a specific agonist leads to attenuation of depressive-like behavior (60, 61). With the aim of developing a GALR2 agonist with higher specificity and greater stability, a new GALR2 specific agonist is developed using SPX as a base structure and substituted with residues of GAL (61). This newly synthesized GALR2 mutant agonist exhibits greater stability in fetal bovine serum compared to the wild-type SPX and possesses the ability to induce anxiolytic effect after i.c.v. injection into the mice. This finding suggests the potential physiological role of SPX in depression and anxiety signaling pathway. Indeed, i.c.v. injection of this agonist in the corticosterone-implanted mice was able to induce antidepressive and anxiolytic effect while restoring the body weight back to a normal level. Furthermore, intranasal application of this agonist produced the same effect as i.c.v. administration, increasing the therapeutic application of this agonist (26).

SPX is downregulated in the hypothalamus but upregulated in the hippocampus (in particular the dentate gyrus) and striatum following treatment with an antidepressant, escitalopram in the rat (62). Furthermore, SPX is down-regulated in the placenta of mothers with antenatal depression but up-regulated in mothers using antidepressant treatment (63). The differential expression in depressed vs. mothers treated with an antidepressant in the placenta (63), is probably due to restoration of SPX expression by the 5-HT system. In addition, SPX is upregulated in the amygdala following treatment with a atypical antipsychotics, chlorpromazine in rats (64). Interestingly, SPX in the medial habenula of mice (26) and its ortholog dorsal habenula in zebrafish (28) provide further evidence on the potential role of SPX in regulating depression and anxiety. As the medial habenula projects to the interpeduncular nucleus, which subsequently projects to the raphe nucleus where the serotonergic neurons are located (65), a potential interaction between SPX and the serotonergic system is plausible. In fact, an increase in the c-fos expression of 5-HT neurons of dorsal raphe nucleus was observed in the corticosterone-implanted mice following the injection of SPX-based GALR2 agonist, indicating a potential modulatory mechanism of SPX on 5-HT neurons (26).

The presence of SPX in the locus coeruleus, hypothalamic paraventricular nuclei (PVN), and the Barrington's nucleus, also suggest the involvement of SPX in stress response and behavior. In the Barrington's nucleus, SPX was found to co-localize with CRH. Although Barrington's nucleus has been known to be involved in the control of micturition, its role in the neural circuitry of stress could not be undermined, due to several factors (66). There are many CRH neurons in the Barrington's nucleus; in fact, it is the primary source of CRH innervation to the core of locus coeruleus, an area which play a prominent role in the neural circuit of stress response (66, 67). Besides, the Barrington's nucleus also received many afferent projections from various brain regions involved in behavioral response including the PVN (68). The presence of SPX in these inter-connected brain regions suggest a possible involvement of SPX in the neural circuit of stress response.

Various studies have provided evidence about the role of GALR2 and GALR3 in the mechanism of depression (60, 69). i.c.v. injection of GALR2 agonist into the rodent decreased the immobility time in the forced swim test. An opposite result was obtained via the injection of GALR1 agonist (70). Additionally, GALR3 antagonist injection in the rat results in anxiolytic and antidepressant properties (69). Activation of GALR1 and GALR3 results in depressive-like phenotype, while stimulation of GALR2 reduces depressive-like behavior (69, 70). This discrepancy in action is the result of activation of a different signaling pathway by different GALR subtypes. GALR1 and GARL3 are connected to inhibitory G proteins G_i_/G_O_, whereas GALR2 activates excitatory signaling mediated through Gq/G_11_ (71, 72). Indeed, SPX display biased agonism toward GALR3 compared with GAL, which may lead to different internalization rate of the receptor and subsequent signaling pathway (73). SPX, acting as a cognate ligand for GALR2/3, could be involved in anxiety/depression. Currently, there are only three studies which demonstrate the biological action of SPX mediated through GALR (10, 24, 46), and the post signaling mechanism of SPX in anxiety/depression which are “pro-depressive” or “anti-depressive.”

## Perspective on Functional Divergence and Conclusion

The coding structure of SPX varies from five to six exons across the vertebrate species. Despite the diversification across species, the 14-amino acid mature peptide sequence is well-conserved across vertebrate. Till date, the functions of SPX are postulated to be mediated through GALR2/3. The functional role of GALR3, which is absent in the fish genome may be acquire by GALR2a or GALR2b. Future studies will have to determine which GALR2 is utilized by SPX for its biological action. The functions of SPX are linked to glucose homeostasis, lipid metabolism, reproduction, and feeding behavior in fish. Another potential role of SPX has been anticipated in stress, anxiety, and depression (74). In addition the functional role of SPX2, which is present in non-mammalian vertebrates (8) warrant future investigation.

## Author Contributions

CL and ML wrote the manuscript. TS and IP edited the manuscript.

### Conflict of Interest Statement

The authors declare that the research was conducted in the absence of any commercial or financial relationships that could be construed as a potential conflict of interest.
